# Factors Associated with Maladaptive Eating Behaviors, Social Anxiety, and Quality of Life in Adults with Celiac Disease

**DOI:** 10.3390/nu13124494

**Published:** 2021-12-15

**Authors:** Anne R. Lee, Benjamin Lebwohl, Jessica Lebovits, Randi L. Wolf, Edward J. Ciaccio, Peter H. R. Green

**Affiliations:** 1 Celiac Disease Center, Columbia University Irving Medical Center, New York, NY 10032, USA; bl114@cumc.columbia.edu (B.L.); jl4877@cumc.columbia.edu (J.L.); ejc6@cumc.columbia.edu (E.J.C.); pg11@cumc.columbia.edu (P.H.R.G.); 2 Teachers College, Columbia University, New York, NY 10027, USA; rlw118@tc.columbia.edu

**Keywords:** celiac disease, maladaptive eating behaviors, social anxiety, quality of life

## Abstract

A gluten-free diet (GFD), which is the only treatment for celiac disease (CeD), is challenging and associated with higher levels of anxiety, disordered eating, and lower quality of life (QOL). We examined various demographic and health factors associated with social anxiety, eating attitudes and behaviors, and QOL. Demographics and health characteristics, QOL, eating attitudes and behaviors, and social anxiety of adults with CeD were acquired using validated measures. The mean scores for QOL, SAQ, and CDFAB were compared across various demographic groups using the Z statistical test. The mean QOL score was 57.8, which is in the moderate range. The social anxiety mean scores were high: 78.82, with 9% meeting the clinical cutoff for social anxiety disorder. Those on a GFD for a short duration had significantly higher SAQ scores (worse anxiety), higher CDFAB scores (worse eating attitudes and behavior), and lower QOL scores. Those aged 23–35 years had lower QOL scores (*p* < 0.003) and higher SAQ scores (*p* < 0.003). Being single (*p* < 0.001) and female (*p* = 0.026) were associated with higher SAQ scores. These findings suggest that the development of targeted interventions to maximize QOL and healthy eating behaviors as well as to minimize anxiety is imperative for some adults with CeD.

## 1. Introduction

Celiac disease (CeD) is a genetically mediated autoimmune disease in which exposure to gluten causes symptoms and destruction of the villous architecture of the small intestine. The damage to intestinal villi results in the malabsorption of nutrients. Other organs of the body are also affected by the immune reaction to gluten. Untreated CeD is associated with bone loss, infertility, neuropathy, and neuropsychiatric symptoms [1,2,3]. Chronic undernutrition has multiple long-term negative effects on physical condition and activities of daily life, and has been well-documented in the literature [4]. The only treatment for CeD to date is lifelong adherence to a gluten-free diet (GFD). Once a GFD is initiated, the intestines often begin to heal, and most individuals report the resolution of symptoms. 

Though symptom improvement can be prompted, a strict GFD must be maintained in the long term. Several studies note the negative impact of the strict nature of the diet. [5,6,7]. In a previous study by our group [5], we found that quality of life (QOL) was significantly impacted in individuals on a GFD. The negative impact was found to be most strongly associated with the social domain of QOL, in particular, dining out, social events, work-related meals, and travel. In a study by Cranney et al. [6], 81% of individuals reported that they no longer dined out, 94% brought their own food when traveling, and 38% avoided travel due to the difficulty of maintaining a GFD. Wolf et al. [7] investigated the association between dietary adherence and QOL. It was found that lower QOL scores were associated with higher vigilance (stricter or more compliant) in adults. However, the participants reported that the restrictive nature of the diet was a daily burden [7]. In a qualitative study by Sverker [8], participants expressed that they were feeling isolated, always thinking about their food, and having concerns over the safety of their food. Since various studies have reported a diminished QOL associated with CeD and a GFD, researchers have determined that the negative impact of CeD and a GFD may have a far-reaching effect, including psychological effects and eating disorders, and may be associated with depression [9,10,11,12]. In a review, Zingone et al. [10] investigated the literature for insight into the potential psychological aspects (anxiety, depression, and fatigue) of the disease and whether treatment with a GFD modified the overall QOL. The results of the literature review indicated that CeD has a significant psychological impact [10], and that anxiety and depression may be ongoing issues in CeD. The researchers felt that anxiety and depression may affect dietary adherence and, therefore, may further influence the overall QOL [10]. 

Recently, several studies have investigated the association between CeD and the social domain of QOL [13,14,15,16]. A study investigating the efficacy of one-on-one counseling versus group counseling sessions found higher anxiety scores in the group-counseled participants compared to the one-on-one category. [14]. In our previous work, individuals with CeD who participated in face-to-face social support systems had higher QOL scores when compared with those who participated only in online support systems [15]. Additionally, the burden on the caregivers and partners of individuals with CeD and following the GFD has been documented [17,18], and the results indicate a wide negative impact that may influence social behaviors. However, the recent research has not investigated the presence of altered social (behavioral) patterns and their association with the rigid nature of the GFD, the burden of dietary adherence, and the constant vigilance needed to avoid chance gluten exposure, especially in social dining situations. In this study, we investigated the demographic and health characteristics associated with social anxiety, eating attitudes and behaviors, and QOL.

## 2. Materials and Methods

This is a cross-sectional study of 538 adults with CeD, investigating the association between demographics and health characteristics, QOL (Celiac Disease Quality of Life (CDQOL)), eating attitudes and behaviors (Celiac Disease Food Attitudes and Behaviors (CD-FAB)), and social anxiety (Social Anxiety Questionnaire).

### 2.1. Recruitment

An email was sent to the entire email distribution list of the Celiac Disease Center. This list consisted of patients, family members, and interested affiliates who opted in to receive communication from the Center. The email inquired whether the receiver was interested in participating in a study to examine the association between CeD, a GFD, and social behavior. The email contained a secure link to the self-administered survey via a web-based survey platform, Qualtrics XM. The initial email invitation was sent on 18 May 2020, with a reminder email sent on 1 June 2020. Of note, this was conducted during the COVID–19 pandemic when the restrictions on dining and travel were in place. The survey closed on 15 June 2020. Response-rate-related numbers are provided below in the Results section.

### 2.2. Inclusion Criteria

The Inclusion criteria were as follows: an age of 18 years or older, a biopsy-proven CeD diagnosis (which was self-reported), and self-reported adherence to a GFD. Preliminary questions on the survey inquired whether the participant met the inclusion criteria. If the participant did not meet the inclusion criteria, the survey was terminated.

### 2.3. Study Measures

We collected the following demographics and health characteristics: gender, marital status, socioeconomic data, level of education, and length of time on a GFD.

The questionnaire included three validated surveys: CD-QOL, SAQ, and CD-FAB. Each of these tools is described below.

CD-QOL-Celiac Disease Quality of Life: The CD-QOL is a 20-item self-report celiac disease specific measure, using a 5-point Likert scale to assess the QOL in adults. The survey generates an overall QOL score, as well as assesses four subdomains of QOL. Scores range from 0 to 100, and higher scores suggest a better QOL (a poor score is less than or equal to a score of 40, and a good overall score is greater than or equal to a score of 60) [19].

Celiac Disease Food Attitudes and Behaviors (CD-FAB): The CD-FAB is a recently developed 11-item self-reported, validated tool that investigates the eating attitudes and behaviors resulting from beliefs concerning cross-contamination, trust, risk-taking, and food safety. Items are based on a 7-point Likert scale. Scores range from 11 to 77, with a higher score suggesting more maladaptive eating attitudes and behaviors [20].

SAQ-Social Anxiety Questionnaire for Adults: The SAQ is a validated 30-item survey using a 5-point Likert scale, reporting on interactions with strangers, speaking in public/talking with people in authority, interactions with the opposite sex, criticism and embarrassment, and assertive expression. The SAQ has five dimensions. The dimensions divide social situations into specific interactions that provide a more detailed indicator of the specific type of social anxiety. Each dimension has different cutoffs for men and women separate from the total score. The five dimensions are speaking in public/talking with people in authority, interactions with the opposite sex (or someone you are attracted to), reaction to assertive behavior, reaction to criticism and embarrassment, and interactions with strangers. The scores range from 30–150. Those with a higher SAQ score have higher levels of social anxiety. Scores greater than or equal to 92 for men and 97 for women meet the clinical cutoff for social anxiety disorder [21].

### 2.4. Statistical Analyses

The demographic and health characteristics of the study population are described by means, frequencies, and percentages. The total score and subscores for each instrument were calculated according to the specific scoring protocol of the instrument. Differences between demographic groups and associations between SAQ, QOL, and CDFAB instruments were analyzed using the Z statistical test. For this test, a two-sided *p*-value of less than 0.05 was considered significant.

### 2.5. Ethical Approval

The Institutional Review Board at Columbia University Medical Center approved this study (AAAS9247) on 22 April 2020. Participants were informed that clicking on the link to the survey and completing it indicated their consent to participate.

## 3. Results

Overall, the response rate was 10.2%, as 13,495 emails were sent to the Celiac Disease Center email list. A total of 5249 emails were opened and 783 (14.9%) recipients participated in the survey. Of the 783 initial participants, 139 did not meet eligibility criteria and 106 partially completed the surveys, resulting in a study population of 538. Table 1 describes the demographic characteristics of the study population. 

### 3.1. Demographics

The study population was predominately women (87.6%), educated at the college and/or graduate school level (78%), and lived in suburban areas (60%). The majority had a household income over USD 100,000 (52%) (Table 1). Most participants were married (59.3%) and many participants reported being on a GFD for ten or more years (38.7%).

### 3.2. Celiac Disease Quality of Life (CD-QOL)

The mean QOL score for the study sample was 57.8 out of 100, corresponding to a moderate QOL. Age, marital status, and length of time on the diet were associated with QOL. Participants aged 23–35 years (compared to greater than 65 years), engaged (compared to all other marital statuses), and adhered to a GFD for less than 1 year (compared to dietary adherence for 10 years or more) had significantly lower QOL scores (*p* < 0.05). The QOL score was significantly improved for those in the older age categories (*p* = 0.003) and trended toward a higher QOL score for those on a GFD for more than 10 years (*p* = 0.12). Of note, there were higher QOL scores for those who identified as married (58.92) compared to those who identified as single (55.54) or engaged (41.39), although this finding was not statistically significant (Table 2).

There were lower QOL scores among those with a lower level of income and education. Those with just a high school education had significantly lower QOL scores compared to those with a college and/or graduate education (*p* = 0.077). A similar significant trend was seen with those who earned USD 20,000–49,999 (*p* = 0.047) compared to those with an income greater than USD 200,000, or those with an income of USD 50,000–74,999 (*p* = 0.131). There was no significant difference between participants from rural, suburban, or urban areas.

### 3.3. Social Anxiety Questionnaire for Adults (SAQ)

The demographic categories of gender, marital status, and age had a significant impact on the SAQ scores, whereas other demographic categories did not. Women had higher scores than men (*p* = 0.026) and participants aged 23–35 compared to those over 65 years, had significantly higher SAQ scores (*p* < 0.003) (Table 2). Of note, not only did the single participants have higher SAQ scores (84.67) than those who identified as married (76.54, *p* < 0.001), but their score approached the clinical cutoff for the diagnosis of a social anxiety disorder (greater than 92 for men and greater than 97 for women). There was no association between the score and education, location, or length of time on the diet.

The subdimensional scores from the specific dimension details of the participants’ reactions to specific types of social interactions are more indicative of overall and specific social anxiety than the total score. Participants who were single and between the ages of 23–35 years scored higher (13.5 and 12.72, respectively) than the other groups in that specific demographic category.

In the dimension of dealing with criticism and embarrassment, both age and sex showed significant differences with a cutoff of 19 for men and 21 for women. Women again scored higher (12.57) indicating more social anxiety than men (8.82). In the category of age, the 23–35-year-olds scored 14.7 (indicating more social anxiety) compared to those over 65 years, who scored 8.95. In the category of marital status, singles scored highest, with a value of 15.96, compared to those who reported being married.

A notable finding concerning the subgroup of participants who reported as being not married (separated, divorced, or widowed), while they did not meet the overall SAQ clinical cutoff for social anxiety disorder, the group did meet the cutoff for the subdimensions of interactions with strangers, reaction to criticism, and embarrassment, as well as talking to people in authority. The total number of participants in this group was only 7.6% of the total study population; therefore, this may be an anomaly rather than a specific trend. However, it is important to note that the subdimensions that participants struggled with are those most closely related to the tasks needed for maintaining a GFD while dining out.

### 3.4. Celiac Disease Food Attitudes and Behaviors (CD-FAB)

The exact cutoff for maladaptive eating patterns has yet to be firmly established with the CDFAB. Based on Satherly’s paper [20], the range (11–77) and median score (33) were used to establish a cutoff (over 49.5) to indicate groups that may be trending toward maladaptive eating behaviors. The only demographic categories that scored beyond this cutoff and were classified as having maladaptive eating behaviors included participants with an income less than USD 20,000 or those who only received a high school education.

Compared to the participants who were over 65 years of age, those in the 23–35 years age group had higher CDFAB scores (*p* = 0.129), suggesting more CeD-specific maladaptive eating attitudes and behaviors, although these differences were not statistically significant. We also found that those that lived in rural areas (48.46), were engaged to be married (48.72), and were on a GFD for one to four years (46.89) had scores that trended toward maladaptive eating attitudes and behaviors; however, these findings were not statistically significant.

QOL scores trended toward improvement, while SAQ scores trended toward worsening with increasing age. The highest QOL and lower SAQ scores were found in the 65+ years age group compared to those in the 23–35 years age group (*p* < 0.001), who had the highest SAQ score and the lowest QOL score for the category. The participants who identified as engaged had the lowest QOL score (41.39) and a moderate SAQ score (79.17), whereas those who identified as single had a moderate QOL score (55.54) and an SAQ score of 84.67, which approaches the cutoff for social anxiety disorder.

**Figure 1:**
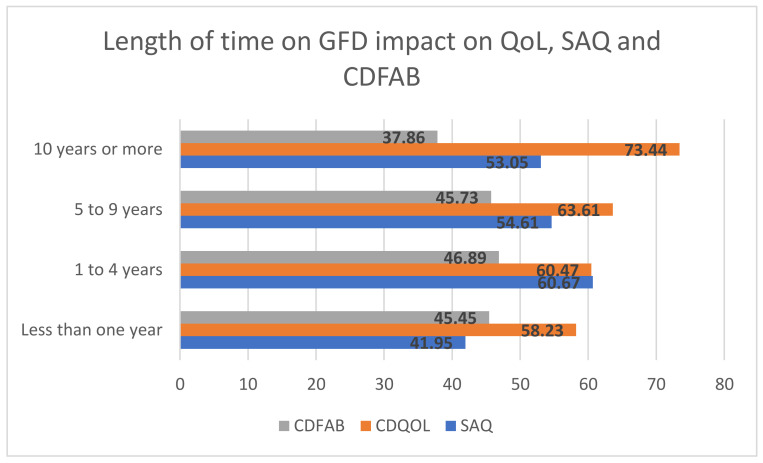
Impact on QOL, social anxiety, and eating behaviors based on the length of time on a GFD.

Similarly, length of time on a GFD was associated with these measures. Compared to those on a GFD for 10 years or more, those on a GFD for one to four years had significantly higher CDFAB scores (i.e., worse eating attitudes and behaviors), lower QOL scores, and higher SAQ scores (i.e., more anxiety) (Figure 1).

## 4. Discussion

The results of this study indicate a concerning impact of the GFD and CeD on patient QOL, social anxiety, and eating attitudes and behaviors. The impact of the GFD and CeD on an individual’s QOL has been well-documented. In previous studies, prior to the COVID -19 pandemic, mean QOL scores ranged from 64.2 to 74.1 [5,7,15]. The mean QOL score for this study was 57.8, potentially reflecting the burden of the pandemic restrictions. The impact of the GFD and CeD on social and eating behaviors had not been previously investigated. This study revealed several trends indicating that young adults (23–35 years of age), those on a GFD for one to four years, and those with a marital status of single or engaged may experience more anxiety, more disordered eating attitudes and beliefs, and a lower QOL compared to others.

Of particular interest are the subdimensions of the SAQ. The five dimensions—speaking in public/talking with people in authority, interactions with the opposite sex (or someone you are attracted to), reaction to assertive behavior, reaction to criticism and embarrassment, and interactions with strangers—identify specific behaviors and provide insight into the impact of living socially while following a GFD. For an individual with CeD, detailing the specific subdimensions is key in determining the link between underlying social anxieties and eating attitudes and behaviors. In previous QOL studies, participants often commented that it was uncomfortable to request special meals, ask to speak to a manager, or request a GF menu, all of which are essential to obtaining a safe GF meal when dining out. Speaking to the wait staff or a manager in a restaurant would correlate to the subdimension of talking to people in authority. The areas of talking with people in authority, reaction to criticism and embarrassment, and interactions with strangers correlate closely to the specific actions required to safeguard an individual with CeD when in social situations. In each of these dimensions, there were similar trends that showed the greatest impact on women versus men, in the 23–35-year-old age group compared to the older participants, and on single individuals. The greatest social anxiety was felt by these demographic groups, which consist of individuals who are potentially at the most socially active time in their life with frequent dining out, embarking on a career, and dating/seeking a partner. This may reflect the increased burden of maintaining a GFD while dating or being without a supportive partner.

We found that those on a GFD for less than one year had lower, but not statistically significant, SAQ and CDFAB scores than the group on the diet for one to four years. This may potentially indicate a “honeymoon effect”, where the burdensome aspects of the GFD are not yet fully appreciated or are masked by the relief of symptom improvement. The negative effects of a GFD on eating patterns and behaviors and social anxiety demonstrates the daily burden of living with CeD on a GFD. The change in scores over time illustrates the need for ongoing expert nutrition counseling that includes strategies for navigating safely on a GFD in the social domain of life.

The study by Lebovits et al. [22] particularly highlights the increased anxiety associated with dating and social interactions. The study found that 68.4% of participants reported that CeD had a major or moderate impact on their dating life. Additionally, almost half (48.4%) reported that they were hesitant to date because of their CeD. In relation to our findings on the SAQ, Lebovits [22] found that 81.3% of participants preferred non-food-related activities for the first several dates, thus avoiding the need to navigate a GFD while dining out. Interestingly, 39.3% of the participants reported being uncomfortable explaining their GFD to wait staff, which is in agreement with the high score found in this study in the subdimension of speaking to people in authority.

In the study of dietary adherence, Wolf et al. [7] highlighted the negative impact of strict dietary adherence on QOL. In a subsequent analysis of the teens in that study [23], it was found that half of the study sample (53.3%) expressed more rigidity (vs. flexibility), avoidance (vs. trust), controlling behavior (vs. confidence), and food preoccupation (vs. awareness) when maintaining a GFD. In addition to scoring higher on the CD-FAB, which suggests maladaptive eating attitudes and behaviors, the teens also had significantly lower QOL scores. It suggests, as this study also found, that increased anxiety, as indicated by the increased SAQ scores associated with dietary adherence, dining with others, and fear of gluten exposure, limit an individual’s ability to socialize and diminishes their overall QOL. As in previous QOL studies, participants often noted not wanting to be the center of attention concerning their meal or disease and that they felt socially isolated or embarrassed by requiring different meals or needs.

### 4.1. Strengths

A major strength of this study is that it is the first to investigate the association between altered social behaviors, social anxiety, maladaptive eating patterns, CeD-specific QOL, and demographic characteristics. The use of diverse validated measures to investigate the intersection of social anxiety, quality of life, and eating behaviors in a population of adults with CeD is another strength of this investigation. Additionally, this study is one of the first to investigate the specific dimension of social anxiety in relation to overall QOL and eating behaviors in the population of individuals with celiac disease.

### 4.2. Limitations

One of the main limitations of this study is the timing, as the survey was distributed during the COVID-19 pandemic, which may have had an impact on all aspects of an individual’s life, in particular social behavior and anxiety. Participants self-reported their CeD diagnosis, which is a limitation of this study, but is not uncommon in online survey research. Another limitation of the study is the small numbers of individuals in some of the subcategories of marital status. While the participants who reported that they were engaged (3.35%) had several significant differences in QOL, SAQ, and CDFAB compared to those who were married, single, or not married, the subcategory population was small and may not be reflective of the general engaged population. A potential confound is the predominance of women participants. However, the prevalence of CeD is approximately 1% of the population worldwide, with about 75% of the population being women, or a 1.3 to 1.5:1 ratio.

## 5. Conclusions

The findings of this study identify the extent of the impact of a GFD on multiple aspects of the daily life of an individual with CeD. It highlights the gaps in the current clinical practice and management of CeD that need to be addressed. Counseling should include a detailed analysis of the individual’s food environment, rather than just providing information on food. Counseling should include queries to help identify and understand the barriers and support systems, feelings of isolation, and the emotional and economic burden of the GFD and anxiety. Potentially, the increased cost and availability of gluten-free foods may factor in some of the demographic categories of income and location. It is imperative to know and understand these barriers and support systems to assist our patients in developing strategies to navigate social situations. This understanding would enable the clinician to address any psychological factors early and potentially prevent the worsening of these impactful clinical situations.

## Figures and Tables

**Table 1:** Study demographics.

Baseline Demographics	
Age (years)	Total: 538
18–22	40 (7.43%)
23–35	134 (24.91%)
36–45	103 (19.14%)
46–55	97 (18.03%)
56–65	88 (16.63%)
Over 65	76 (14.13%)
Gender	
Male	67 (12.45%)
Female	471 (87.55%)
Non-binary	0 (0.0%)
Prefer not to say	0 (0.0%)
Highest education level	
Some high school	1 (0.19%)
Graduated high school	27 (5.02%)
Some college	89 (16.54%)
Graduated college	195 (36.25%)
Some graduate school or more	226 (42.01%)
Household Income (USD)	
Less than 20,000	21 (3.9%)
20,000–49,999	66 (12.7%)
50,000–74,999	85 (15.8%)
75,000–99,999	84 (15.61%)
100,000–200,000	169 (31.41%)
Greater than 200,000	113 (21.0%)
Residence	
Urban	161 (29.93%)
Suburban	323 (60.04%)
Rural	54 (10.04%)
Current marital status	
Single	160 (29.74%)
Engaged	18 (3.35%)
Married	319 (59.29%)
Not married	41 (7.62%)

**Table 2:** QOL, SAQ, and CDFAB scores by demographics.

Demographic Category		*N*	CDQOL	SAQ > 92 = Cut off for Anxious	CDFAB >49.5 = Maladaptive <25.5 = Adaptive
Sex	Male	67	59.53	67.7	40.88
	Female	471	57.59	80.4 *	43.39
Age (years)	18–22	40	57.1	85.36	42.28
	23–35	134	50.52	86.56 *	45.75
	36–45	103	52.28	79.32	45.51
	46–55	97	60.40	75.5 *	43.89
	56–65	88	60.20	75.26 *	43.47
	Over 65	76	72.60 *	68.57 *	33.99
Education	Graduated high school	27	40.97	87.04	51.04
	Some college	89	57.76	81.27	43.33
	Graduated college	195	56.97	80.31	42.61
	Some graduate school or more	226	60.76	75.29	42.31
Income (USD)	Less than 20,000	21	41.67	89.57	54.14
	20,000–49,999	66	48.49	85.32	46.94
	50,000–74,999	85	53.19	81.20	46.18
	75,000–99,999	84	57.32	78.73	43.6
	100,000–200,000	169	61.41	75.46	42.01
	Greater than 200,000	113	64.84 *	75.92	37.64
Location	Urban	161	59.91	76.46	41.52
	Suburban	323	57.86	79.08	42.95
	Rural	54	51.48	83.68	48.46
Marital status	Single	160	55.54	84.67	43.31
	Engaged	18	41.39	79.17	48.72
	Married	319	58.92	76.54	43.37
	Not married (separated/divorced/widowed)	41	65.61	72.53	36.98
Length of time on a GFD	Less than one year	22	47.78	80.09	45.45
	1 to 4 years	163	50.59	80.78	46.89
	5 to 9 years	145	54.51	80.04	45.73
	10 years or more	208	66.8 *	76.22	37.98

* denotes significance < 0.05.

## Data Availability

The data presented in this study are available on request from the corresponding author. The data are not publicly available due to participant privacy.
